# Robinow syndrome

**DOI:** 10.4103/0019-5413.43399

**Published:** 2008

**Authors:** SS Suresh

**Affiliations:** Department of Orthopaedics Ibri Regional Referral Hospital, Ibri, Sultanate of Oman

**Keywords:** Dwarfism, Robinow syndrome, scoliosis

## Abstract

Robinow syndrome is a rare autosomal recessive mesomelic dwarfism with just more than 100 cases reported in the literature so far. The lower extremity is spared with skeletal deformity usually confined to the forearm, hand, and the dorsal spine. Diagnosis is made easily in the early childhood by the typical “fetal facies” appearance, which disappears to a certain extent as the patient grows. The author reports two cases of this entity with vertebral segmentation defects, rib fusion, and typical severe brachymelia and facial features.

## INTRODUCTION

Robinow syndrome is a rare form of mesomelic dwarfism, which is reported from the Arab countries, Czechoslovakia, and also from Indian subcontinent.^1^–^3^ There is a resemblance to the fetal face at birth with laterally displaced eyes, forward pointing ala of nose, and broad and prominent forehead, and hence, Robinow named this as “fetal facies” syndrome.^1^ The recessive form is easily diagnosed when present with thoracic vertebral segmentation defects and rib anomalies in association with typical facies and genital abnormalities. Diagnosis is easy if the typical clinical features and the radiological findings are known.

## CASE REPORT

Two boys of almost similar age born in 1990 were brought to the orthopedic clinic for disability assessment for social security. Both were unrelated and born to consanguineous parents. The skeletal features were strikingly similar.

### Case 1

**Figure 1:**
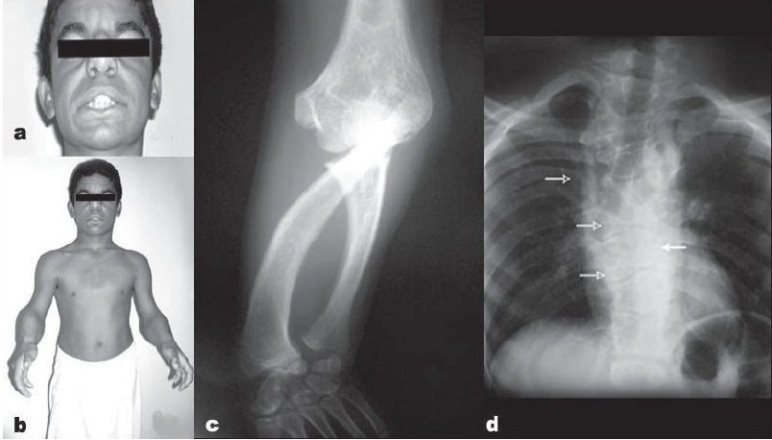
Case 1 (a) Facial features of Robinow syndrome showing broad forehead, hypertelorism, and broad and upturned nose. (b) Profile of patient showing normal upper arm, elbow deformity, and short forearm. (c) Anteroposterior X-ray of forearm with elbow showing dislocated radial head, bowed radius, and hypoplastic ulna (noted bilaterally). (d) Chest X-ray film showing rib anomaly (white arrow), hemivertebrae (white arrows), and block vertebra (bold arrow)

The patient was short for his age with normal intelligence. His parents and other siblings in the family were normal [Figure 1(a,b)]. He had mesomelic shortening of the upper limbs, with dislocated radial head and bowing of the shaft of the radius. The ulna was short compared with the radius. He had practically no rotations of the forearm [Figure 1c]. The humerus was normal. Other features observed were hypertelorism and broad and prominent forehead. The nose was upturned with anteverted nares and long philtrum. The lower limbs were comparatively normal, and the radiographs of the pelvis and knees were also normal. He had scoliosis of the dorsal spine with multiple hemivertebrae and block vertebrae. Ribs were abnormal at multiple levels with fusion of the ribs at the costochondral junctions [Figure 1d]. He was previously treated for undescended testis and had micropenis and features of hypovirulization.

### Case 2

**Figure 2:**
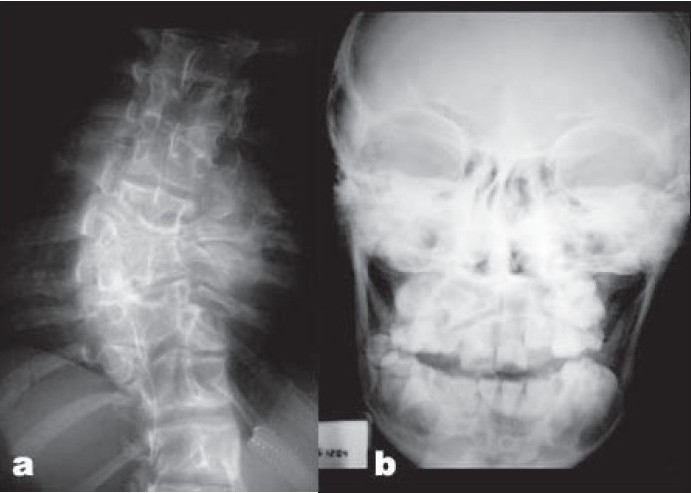
Case 2 (a) Dorsal spine X-ray film showing scoliosis, block vertebrae and hemivertebrae. (b) Skull X-ray film showing crowded teeth

The skeletal features in this patient were strikingly similar except that the scoliosis was of much severe degree [Figure 2a]. He also had the typical forearm deformity and costovertebral segmentation defects. This patient underwent functional endoscopic sinus surgery for antrochoanal polyp. His teeth were crowded [Figure 2b] and had gingival hypertrophy. His ears as in the previous case were low set, and the pinna was deformed.

## DISCUSSION

Robinow syndrome described in 1969 by Meinhard Robinow is a rare genetic disorder characterized by short stature and abnormalities in the head, face, and external genitalia associated with vertebral segmentation defects. More than 100 cases have been reported in the literature. Both autosomal dominant and recessive modes of inheritance are known, with the recessive form having more severe symptomology. This syndrome is also known as “fetal facies” syndrome as the facial features of infants with this syndrome resemble those of an 8-week-old fetus.

The incidence is reported to be one in every 500,000 people with equal frequency in male and female. The prevalence is low, as 5–10% of the children die in infancy because of cardiac problems.^4^ Autosomal recessive form has been reported in Oman because of high degree of consanguineous marriages^1^,^5^ and hemoglobinopathies.^6^ A total of 12 cases have been reported in Oman^6^. The frequency of Robinow syndrome is reportedly high in Turkey.^7^

Diagnosis depends on the clinical features, but clinicians and radiologists should be familiar with the radiological features that are seen in moderate or severe cases of Robinow syndrome.^8^ All patients in the recessive form suffer from vertebral segmentation abnormalities, resulting in kyphoscoliosis and chest deformities. Thoracic vertebrae are commonly fused with frequent hemivertebrae; hence, this anomaly was previously known as COVESDEM (costovertebral segmentation defect with mesomelia) syndrome. Ribs are also commonly deformed. Hemivertebrae and scoliosis were present in more than 75% of patients with the recessive form, but in less than 25% of patients with the dominant form.^9^ Mazzeu clinically categorized the patients into recessive and dominant forms based on the presence of rib fusions.

There is midfacial hypoplasia with a short upturned nose. Patients have broad and prominent forehead. Children have hypertelorism. Upper lip usually has an inverted “V” appearance, thus exposing the incisors and upper gum. Usually gum hypertrophy is present from birth. Teeth are crowded and irregular. Ears are low set, and the pinna is deformed. About 10% of children have an early fatal outcome because of congenital heart disease, with commonest abnormality reported to be pulmonary stenosis and atresia.^1^

Soliman *et al*, reported empty sella in all 12 patients analyzed by them in Oman.^10^ There is a low basal testosterone level with a defective sex steroid feedback mechanism. There is mesomelic shortening of the extremities, with dislocation of the radial head with limitation of forearm rotations. There are abnormalities in the hands and fingers also with shortening of the distal phalanges. Madelung deformity also has been reported. There is severe distal ulnar and proximal radius hypoplasia, with the radial head dislocated. Lower extremities are usually less affected.

In patients with almost normal skeletal survey, fetal facies, genital hypoplasia, and gingival hyperplasia with crowded teeth are the three major dysmorphic features that are diagnostic.^8^ Micropenis is a constant feature in Robinow syndrome. In females, the clitoris is reduced in size with underdeveloped labia minora. Soliman reported endocrine dysfunction and described empty sella as a feature of Robinow syndrome.^10^

The causative gene is *ROR2* on position 9 of the long arm of chromosome 9. This gene is responsible for bone and cartilage growth. Prenatal diagnosis is possible from the 19th week of pregnancy by fetal ultrasonography. Assessment of the length of the long bones and the ulna/humerus ratio has been used in prenatal diagnosis.^1^,^11^

This syndrome has to be differentiated from other causes of vertebral malsegmentation, rib hypoplasia, and forearm dysplasias. These are differentiated by the characteristic facial changes and the genital hypoplasia. Children are more prone to recurrent urinary tract infections due to hydronephrosis. Renal tract abnormalities and cystic dysplasia of the kidneys have been documented.^1^

Prognosis of Robinow syndrome is generally good, with more than 80% patients having normal intelligence. Management of the skeletal deformities includes bracing or surgical correction of scoliosis. The forearm deformities are correctable to a certain extent by callotaxis with a ring fixator. Recombinant human growth hormone is known to increase the growth rate of children with the coexisting growth hormone deficiency.^4^ There are also reports of treatment with human chorionic gonadotrophin to increase the penile length and testicular volume.^3^

## References

[1] Patton MA, Afzal AR (2002). Robinow syndrome. J Med Genet.

[2] Kulkarni ML, Reddy S (2004). Images in clinical practice: Robinow Syndrome. Indian Pediatr.

[3] Centre for Arab Genomic Studies Robinow syndrome, Autosomal Recessive.

[4] Hosalkar HS, Shaw GJ (2002). Robinow syndrome. J Postgrad Med.

[5] Afzal RA, Rajab A, Fenske CD, Oldridge M, Elanko N, Periera ET (2000). Recessive Robinow syndrome, allelic to dominant brachydactyly type B, is caused by mutation of ROR2. Nat Genet.

[6] Rajab A, Bappal B, Al-Shaikh H, Al-Khusaibi S, Mohammed AJ (2005). Common autosomal recessive diseases in oman derived from a hospital- based registry. Community Genet.

[7] Aksit S, Aydinlioglu H, Dizdarer G, Caglayan S, Bektaslar D, Clin A (1997). Is the frequency of Robinow syndrome relatively high in Turkey? Four more case reports. Clin Genet.

[8] Kaissi AA, Bieganski T, Baranska D, Chehida FB, Gharbi H, Ghachem MB (2007). Robinow syndrome: Report of two cases and review of the literature. Austr Radiol.

[9] Mazzeu JF, Pardono E, Vianna-Morgante AM (2007). Clinical characterization of autosomal dominant and recessive variants of Robinow syndrome. Am J Med Genet Am.

[10] Soliman AT, Rajab A, Alsalmi I, Bedair SMA (1998). Recessive Robinow syndrome: With emphasis on endocrine functions. Metabolism.

[11] Guven MA, Batukan C, Ceylaner S, Uzel M, Ozbek A, Demirpolat G (2006). Prenatal and postnatal findings in a case with autosomal recessive type of Robinow syndrome. Fetal Diagn Ther.

